# HTLV-1 HBZ cooperates with JunD to enhance transcription of the human telomerase reverse transcriptase gene (hTERT)

**DOI:** 10.1186/1742-4690-4-92

**Published:** 2007-12-13

**Authors:** Anne-Sophie Kuhlmann, Julien Villaudy, Louis Gazzolo, Marc Castellazzi, Jean-Michel Mesnard, Madeleine Duc Dodon

**Affiliations:** 1 Virologie Humaine, INSERM-U758, 69364 Lyon Cedex 07, France; 2 Ecole Normale Supérieure de Lyon, 69364 Lyon Cedex 07, France; 3 IFR 128 BioSciences Lyon-Gerland, 69364 Lyon Cedex 07, France; 4 Laboratoire des Infections Rétrovirales et Signalisation Cellulaire, CNRS/UM I UMR 5121/IFR122, Institut de Biologie, 34960 Cedex 2 Montpellier, France

## Abstract

**Background:**

Activation of telomerase is a critical and late event in tumor progression. Thus, in patients with adult-T cell leukaemia (ATL), an HTLV-1 (Human T cell Leukaemia virus type 1)-associated disease, leukemic cells display a high telomerase activity, mainly through transcriptional up-regulation of the human telomerase catalytic subunit (hTERT). The HBZ (HTLV-1 bZIP) protein coded by the minus strand of HTLV-1 genome and expressed in ATL cells has been shown to increase the transcriptional activity of JunD, an AP-1 protein. The presence of several AP-1 binding sites in the hTERT promoter led us to investigate whether HBZ regulates hTERT gene transcription.

**Results:**

Here, we demonstrate using co-transfection assays that HBZ in association with JunD activates the hTERT promoter. Interestingly, the -378/+1 proximal region, which does not contain any AP-1 site was found to be responsible for this activation. Furthermore, an increase of hTERT transcripts was observed in cells co-expressing HBZ and JunD. Chromatin immunoprecipitation (ChIP) assays revealed that HBZ, and JunD coexist in the same DNA-protein complex at the proximal region of hTERT promoter. Finally, we provide evidence that HBZ/JunD heterodimers interact with Sp1 transcription factors and that activation of hTERT transcription by these heterodimers is mediated through GC-rich binding sites for Sp1 present in the proximal sequences of the hTERT promoter.

**Conclusion:**

These observations establish for the first time that HBZ by intervening in the re-activation of telomerase, may contribute to the development and maintenance of the leukemic process.

## Introduction

Adult T-cell leukaemia (ATL) is a T-cell malignancy that develops in about 5% of asymptomatic HTLV-1 (human T-cell leukaemia virus, type 1) carriers after a latent period ranging from 20 to 60 years, indicating a multistage process of transformation of T lymphocytes. ATL cells are generally CD4+ T lymphocytes, in which both NF-κB and AP-1 (activator protein-1) transcription factors are constitutively active. Distinct clinical subtypes of ATL include two indolent forms, smoldering and chronic, and extremely aggressive forms, acute and lymphomatous. Chronic ATL often progresses to acute or lymphoma-type ATL and the mean survival time of patients with acute ATL is about one year [1-3]. Interestingly, the close correlation observed between telomerase activity and the clinical stage of the disease indicates that the re-activation of telomerase, by contributing to telomere stabilization, is a key event in development and progression of ATL [4].

A functional basic leucine zipper (bZIP) protein, HBZ (HTLV-1 bZIP factor), that is encoded by a mRNA transcribed from a functional promoter present within the anti-sense strand of the 3' end of the HTLV-1 provirus, was identified, through its expression in several HTLV-1-infected cell lines [5-7]. Moreover, HBZ was found to be the only viral gene product detected in a panel of fresh ATL cell clones [8]. This protein contains an N-terminal transcriptional activation domain, two basic regions corresponding to nuclear localization signals, and a DNA-binding domain upstream of a C-terminal leucine zipper motif [9,10]. Interestingly, HBZ RNA was found to promote T-cell proliferation and to up-regulate the E2F1 transcription factor [8]. Furthermore, the HBZ protein has been shown to interact with other bZIP proteins, in particular with the AP-1 transcription factors, resulting in the modulation of their transcriptional activity [11-13]. Thus, through its interaction with CREB-2 (also called ATF-4), HBZ inhibits Tax-mediated proviral transcription from the HTLV-1 promoter within the viral LTR [10,14-16]. Tax, a viral regulatory protein, encoded by the pX region of HTLV-1, plays a pivotal role in the early steps of the transformation of T lymphocytes infected by HTLV-1, by influencing the transcription of numerous cellular genes, among them NF-κB and AP-1 [17-19].

The hTERT proximal core promoter which contains Sp1 and c-Myc binding sites, is essential for the transcriptional activation of this cellular gene [20-22]. Recently, five putative binding sites for AP-1 have been identified within the distal regulatory sequences of the hTERT promoter [23]. AP-1 is composed of heterodimers of Jun (c-Jun, JunB or JunD) and Fos (c-Fos, Fra1, Fra2, FosB-2) proteins and c-Fos/c-Jun and c-Fos/JunD heterodimers have been shown to decrease hTERT transcription in human cells [23]. Interestingly, HBZ is not able to form stable homodimers and is therefore dependent on heterodimerization with other AP-1 proteins to control gene transcription [11-13]. In the present study, we investigated whether HBZ, in association with c-Jun or JunD, is able to regulate the activity of the hTERT promoter. We demonstrated that HBZ together with JunD synergistically activates hTERT transcription through their recruitment by the Sp1 transcription factors on the Sp1 sites present at the proximal region of the hTERT promoter. These observations provide an original insight by which hTERT transcription is up-regulated by this viral protein.

## Results

### HBZ regulates the activity of the hTERT promoter

**Figure 1:**
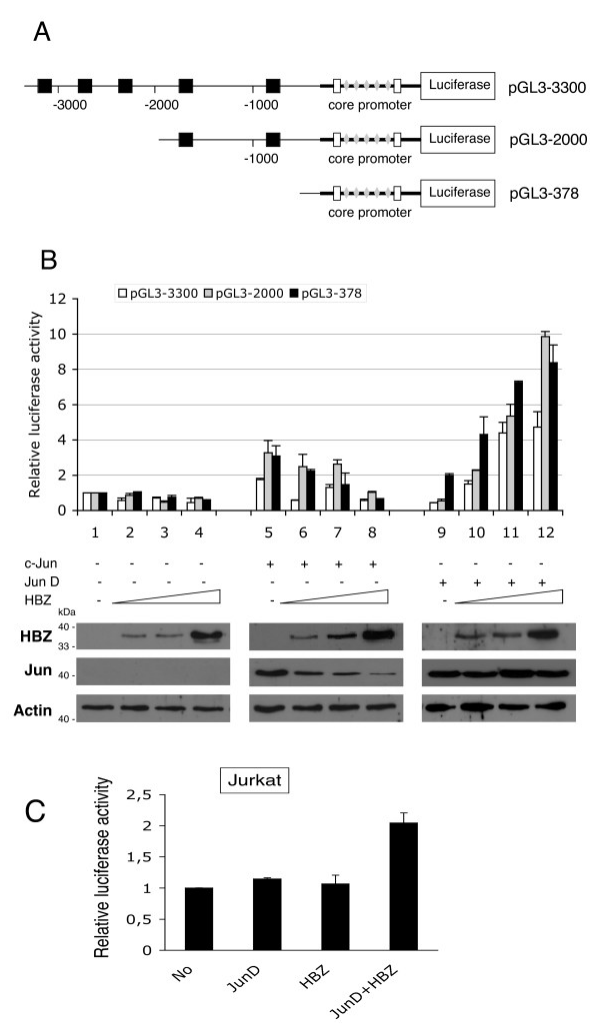
Transient-expression assays to examine the role of HBZ and of AP-1 in the hTERT promoter. (A) Schematic diagram of the luciferase reporter plasmids containing various lengths of the hTERT promoter. The black squares indicate AP-1 responsive sites. The sequence of the proximal core promoter is located between -181 to +80. (B) Effect of HBZ and AP-1 on luciferase reporter constructs. HeLa cells were cotransfected with various lengths of the hTERT promoter plasmids (0.1 μg) and with HBZ- (0.2 to 0.8 μg), and/or c-Jun- (0.2 μg), and/or JunD (0.2 μg)-expression vectors. Luciferase activity was normalized to tk-luc activity and presented relative to cells transfected with the reporter plasmid alone. The values are those obtained in triplicate, from three different experiments. Error bars indicate standard deviations. Shown in the lower panel, a western blot analysis of HBZ and Jun protein levels in whole cell lysates of HeLa samples transfected with pGL3-378. The membrane was probed successively with a polyclonal anti-HBZ antibody, and a mouse anti-flag antibody. Actin was used as a loading control. (C) Transactivation of the hTERT promoter by HBZ and JunD in Jurkat cells. Cells were cotransfected with pGL3-378 reporter plasmid (4 μg), in combination with the indicated HBZ (2 μg) and/or JunD (2 μg)-expression vectors. Luciferase activity was normalized and presented as indicated in B. The values are those obtained in triplicate, from one representative experiment.

To examine the role of HBZ in regulating the activity of the hTERT promoter, luciferase assays were performed with reporter plasmids containing various lengths of the 5' flanking sequence of the *hTERT *gene fused to the luciferase reporter gene (Fig 1A). The longest reporter pGL3-3300 contains 5 AP-1 binding sites; pGL3-2000 includes two of these sites, whereas the shortest construct pGL3-378 encompassing the proximal region is devoid of any AP-1 binding sequence. Each of these reporter plasmids was co-transfected in HeLa cells along with increasing amounts of an HBZ vector either alone or together with c-Jun or JunD expression plasmids. The expression levels of HBZ and Jun proteins were confirmed by Western blot analysis. Overexpression of HBZ with each of the three constructs did not exert any effect on the hTERT promoter activity (Fig 1B, lanes 2, 3, 4). Overexpression of c-Jun or JunD led to a small, but significant increase of this promoter activity. In presence of c-Jun, a 2-fold increase was observed with pGL3-3300, and a 3-fold increase with pGL3-2000 and pGL3-378. In presence of JunD, a 2-fold-increase was observed only with pGL3-378. Overexpression of HBZ with c-Jun resulted in a reduction of the hTERT promoter activity with the three reporter constructs (compare lanes 6, 7, 8 to lane 5). To note that the increased amounts of HBZ correlated with a decrease of c-Jun detected in cell lysates, as previously shown [12]. Intriguingly, overexpression of HBZ in the presence of JunD led to an increase of the hTERT promoter activity, which also correlated with the transfected amount of HBZ (compare lanes 10,11,12 to lane 9). Taken together, these observations show that HBZ expressed either with c-Jun or JunD is able to repress or enhance the hTERT promoter activity, respectively.

Interestingly, the activation observed in presence of JunD was found to be equally high using reporter pGL3-378 and pGL3-2000 constructs. As no AP-1 binding site was present in pGL3-378, this observation suggests that HBZ exerts an indirect control on the hTERT core promoter. Indeed, our data propose that the up-regulation of hTERT promoter activity is mediated by HBZ in cooperation with JunD and indicate that the proximal region of the promoter contains the responsive sequences necessary and sufficient to increase this activity. To confirm the effect of the *HBZ *gene in T cells, we co-transfected Jurkat T cells with the pGL3-378 reporter construct with either JunD or both JunD and HBZ (Fig 1C). It was observed that HBZ, in presence of JunD, increases the hTERT promoter activity, but to an extent lower than that observed in HeLa cells.

### Both the N-terminal and leucine-zipper regions of HBZ are required to increase the hTERT promoter activity

**Figure 2:**
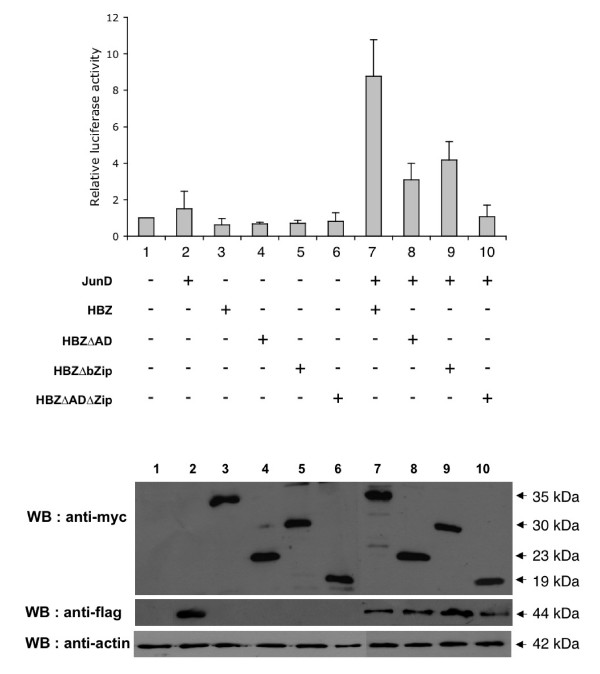
Deletion of the activation domain and leucine-zipper region of HBZ significantly reduces hTERT promoter activity. The luciferase reporter plasmid, pGL3-378 (100 ng) was cotransfected with expression plasmids (200 ng) for JunD, HBZ or HBZΔAD or HBZΔbZIP, or HBZΔADΔZip as indicated. Luciferase activity was normalized to tk-luc activity and is presented relative to that of cells transfected with the reporter plasmid alone. The values are those obtained in duplicate from five experiments. Lower panel: western blot analysis of HBZ and JunD protein levels in whole cell lysates of HeLa samples. The membrane was successively probed with monoclonal anti-c-myc, anti-Flag and anti-actin antibodies.

To identify the domain(s) of the HBZ protein required for transactivation of the hTERT promoter, a structure-function analysis was first performed including vectors expressing three deleted forms of HBZ. The first one, referred to as HBZΔAD, is deleted of the N-terminal 80 amino acids corresponding to the activation domain (AD), and was found to be unable to increase JunD-mediated transactivation of a synthetic collagenase AP-1-Luc construct [13]. The second one, HBZΔbZIP, lacks the 74aa C-terminal domain, which includes the leucine zipper domain, and is therefore unable to form dimers with other AP-1 proteins [11]. The third one, HBZΔADΔZip, lacks both the 80 aa N-terminal domain and the 46aa Zip domain. The wild type (wt) HBZ or each of its deleted forms was transiently co-transfected into HeLa cells along with the pGL3-378 construct, in the presence or absence of the JunD expression vector. Luciferase assays show that the overexpression of wtHBZ in the presence of JunD resulted in a 5.8-fold transactivation of the hTERT core promoter (Fig 2, lane 7). In cells transfected with HBZ mutants deleted either of the activation domain or of the leucine zipper domain, a decrease of this transactivation by 66% and 53% was observed respectively, suggesting that they still retain enhancing activities (lanes 8 and 9). Finally, the double mutant was found to be unable to increase JunD-mediated transactivation (lane 10). Indeed, these results show that both the N-terminal and the C-terminal domains of HBZ are required to fully transactivate the hTERT promoter in presence of JunD. Thus, the activation functions together with the dimerization properties of HBZ appear to be essential for up-regulating the hTERT promoter activity.

### HBZ positively regulates hTERT transcription in presence of JunD

**Figure 3:**
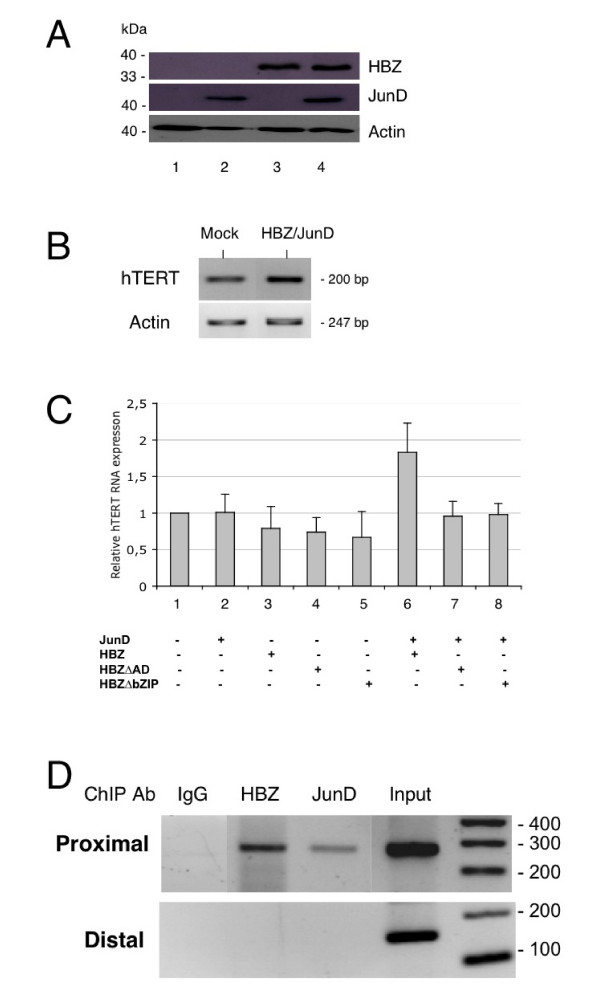
Up-regulation of the hTERT gene transcription in cells overexpressing HBZ and JunD. HeLa cells were cotransfected with expression plasmids for HBZ and JunD and incubated for 48 hours. (A) Western blot analysis of cell lysates using appropriate antibodies; lane 1, mock transfected; lane 2, JunD-transfected; lane 3, HBZ-transfected; lane 4, HBZ/JunD-transfected. (B) RT-PCR analysis of mRNA extracted from transfected cells. The RNAs were isolated and reverse transcribed. PCR was performed using primers specific for hTERT and actin. (C) Real-time PCR quantification of hTERT mRNA expression from cells transfected with indicated plasmids was performed as described in "Materials and methods". The expression level in mock-transfected cells was defined as 1.0. Experimental variations are indicated by error bars. (D) Recruitment of HBZ and JunD to the hTERT proximal promoter by chromatin immunoprecipitation assay (ChIP) in HeLa cells overexpressing HBZ and JunD. PCR results from IP reactions using preimmune rabbit serum (IgG) and antibodies against HBZ and JunD are shown. Each panel shows amplification of 0.4% of the total input chromatin (input). Purified DNA was analyzed by PCR using primers spanning the hTERT proximal promoter (upper panel) or the hTERT distal promoter (lower panel). DNA size standards are indicated. Data are shown for one representative experiment from three independent assays.

The above results propose for the first time that HBZ, in cooperation with JunD, activates the transcription of the hTERT gene. To examine the effect of HBZ on *hTERT *transcription, HeLa cells were co-transfected either with JunD and HBZ expression vectors or a blank vector. A Western blotting analysis, performed 48 hours later, confirmed the expression of both HBZ and JunD proteins (Fig 3A). The level of hTERT transcription evaluated by RT-PCR analysis showed a slight increase of hTERT transcripts, when HBZ and JunD were overexpressed (Fig 3B). Likewise, a quantitative analysis of hTERT mRNAs using real-time PCR revealed a significant 1.8-fold increase (P < 0.05) of hTERT mRNAs in cells co-expressing HBZ and JunD (Fig 3C, lane 6). Such an increase was not observed in cells expressing either JunD alone (lane 2) or HBZ alone (lane 3) or JunD together with one of the mutated forms of HBZ (lanes 7 and 8). These findings demonstrate that HBZ acts synergistically with JunD to increase the transcription of the *hTERT *gene.

To verify that HBZ plays a direct molecular role in the activation of hTERT expression, chromatin immunoprecipitation (ChIP) assays were done to seek evidence of HBZ occupancy at the hTERT promoter. HeLa cells overexpressing HBZ and JunD were crosslinked, sonicated, DNA-protein complexes collected by centrifugation, and ChIP performed (Fig 3D). Both HBZ and JunD were present at the hTERT proximal promoter region. Notably, the specificity of HBZ and JunD ChIP was illustrated by the lack of occupancy at the distal region of hTERT promoter. Collectively, these results confirm that HBZ behaves as a positive regulator of hTERT gene transcription.

### Identification of the promoter sequences responsible for the HBZ/JunD-mediated transcriptional upregulation of hTERT

**Figure 4:**
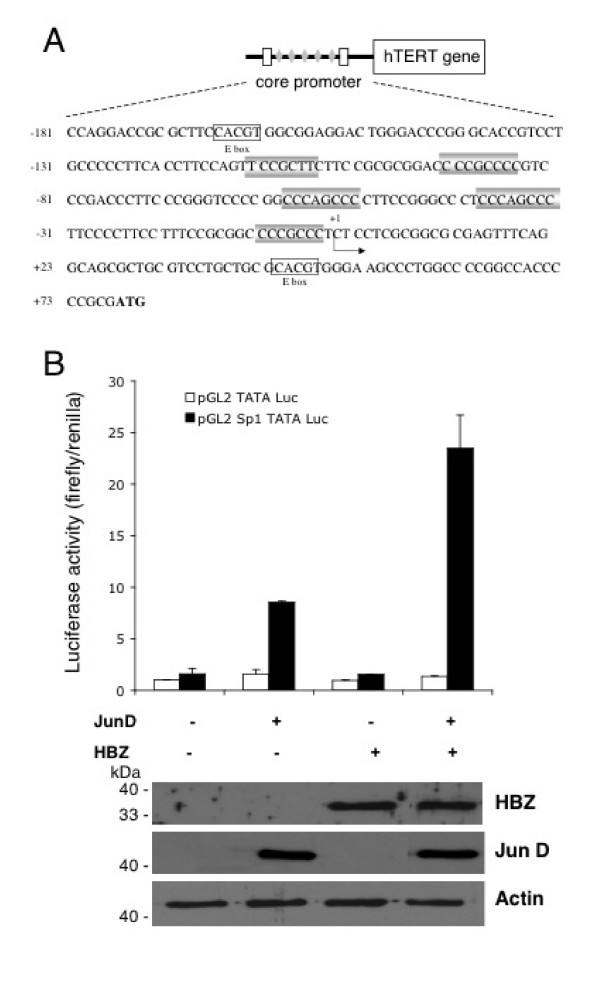
Involvement of Sp1 binding sites in HBZ/JunD-mediated transcriptional activity. (A) Schematic diagram of the hTERT gene promoter corresponding to the sequence of the proximal core promoter (-181 to +80) upstream of the initiating ATG shown in bold. The Sp1-binding sites (shaded box) and E-boxes (white box) are indicated. (B) The luciferase reporter plasmid, pGL2-Sp1-TATA-Luc or the control plasmid, pGL2-TATA-Luc (100 ng) was cotransfected with expression plasmids for HBZ (200 ng) and/or for JunD (200 ng), as indicated. Luciferase activity was normalized to tk-luc activity. Results represent duplicate samples from two different experiments. Lower panel: western blot analysis of HBZ and JunD protein levels in whole cell lysates of HeLa samples transfected with pGL2-Sp1-TATA-Luc plasmid. The membrane was successively probed with a rabbit polyclonal anti-HBZ serum, mouse monoclonal anti-Flag and rabbit anti-actin antibodies.

As demonstrated above, the proximal hTERT promoter is responsible for stimulating the transcriptional activity of the *hTERT *gene by HBZ together with JunD. However, the inspection of the nucleotide sequence of this region does not reveal any sequence homologous to the consensus AP-1 binding site (5'-TGAC/GTCA-3'). This region includes the 180 bp core promoter that contains 2 E-boxes and 5 GC boxes, which represent the consensus binding sequences for Myc/Max and Sp1, respectively (Fig 4A). Interestingly, previous studies have revealed that each of the five Sp1 sites cooperatively function as a *cis*-acting element [21]. The presence of a unique Sp1 site in a 32 bp minimal promoter region suggests that Sp1 may recruit basal transcription factors for hTERT. Furthermore, Jun proteins (and among them JunD) are able to interact with Sp1 proteins [24,25]. We therefore hypothesize that a mechanism involving protein-protein interactions between HBZ and JunD is operative for the transactivation of the hTERT promoter, Sp1 being implicated in the recruitment of the HBZ and JunD to the GC boxes. To verify this hypothesis, a construct, pGL2-Sp1-TATA-Luc, containing a minimal promoter with one Sp1 binding site with a sequence (C_4_GC_4_,) similar to that of the second and fifth Sp1 sites present on the proximal region of the hTERT promoter followed by a TATA box fused to a luciferase reporter gene was co-transfected in HeLa cells together with HBZ- or/and JunD-expression vectors. An identical reporter construct, but lacking the GC box, was used as control. The expression levels of JunD and HBZ proteins were verified by western blotting, 48 h after transfection. In cells cotransfected with the control construct, no significant increase of luciferase activity was observed in presence of either JunD, or HBZ or both (Fig 4B). In HeLa cells transfected with the reporter construct containing the GC-rich Sp1 binding site, the expression of JunD led to a 5-fold increase of the luciferase activity (compared to that of cells transfected with the reporter construct alone), whereas that of HBZ did not exert any effect. The co-expression of JunD and HBZ led to a 15-fold increase of luciferase activity. This observation therefore underlines the intervention of the Sp1 binding sites in the synergistic activation of the hTERT core promoter by HBZ and JunD.

### Physical and functional interactions of HBZ, JunD and Sp1 proteins

**Figure 5:**
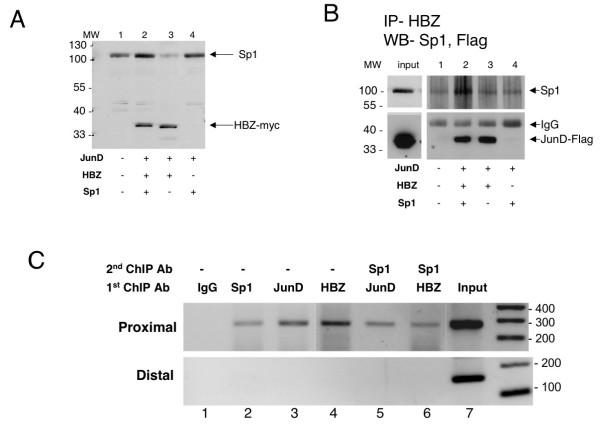
Physical interactions of HBZ, JunD and Sp1 proteins. (A) HBZ and Sp1 expression in transfected HeLa cells by Western blot analysis. (B) *In vivo *interactions detected by Immunoprecipitation and western blot analysis. Five percent (for Sp1) and 0.2% (for JunD) of the whole cell lysate were used as input; lysates (400 μg) were immunoprecipitated as indicated in material and methods with a polyclonal anti-HBZ antibody. (C) HBZ, JunD and Sp1 are tethered to hTERT proximal promoter region. Sequential ChIP assays were performed after an initial IP with either anti-JunD or anti-HBZ. Protein-DNA complexes were detected at the hTERT proximal promoter region, after second IP with anti-Sp1, but not in the distal region. Each panel shows amplification of 0.4% of the total input chromatin (input).

To confirm the *in vivo *interaction of these three proteins, extracts of HeLa cells co-expressing HBZ, JunD and Sp1 were immuno-precipitated with a rabbit anti-HBZ serum. Proteins in the immuno-precipitate were then analyzed by western blot using a monoclonal anti-Flag antibody or a rabbit anti-Sp1 serum. Under these experimental conditions, JunD was found in the immunoprecipitate only when HBZ is expressed (Fig 5B, lower panel lanes 2 and 3). When the same experiment was performed with extracts from HeLa cells either mock-transfected or transfected with JunD and Sp1, these proteins were not detected in the immunoprecipitate (Fig 5B, lower panel lanes 1 and 4), confirming the specificity of the association between HBZ and JunD. In addition, Sp1 was found to be more abundant in the immunoprecipitate prepared from HeLa cells overexpressing Sp1, JunD and HBZ (Fig 5B, upper panel, lane 2), underlining the interaction of the three proteins.

To demonstrate that HBZ, JunD and Sp1 co-exist within the same protein complex resident in the proximal promoter, sequential ChIP assays were performed (Fig 5C). In such assays, an initial ChIP was performed with an antibody that recognizes one protein. The precipitated chromatin-DNA complex was washed and eluted, then a second IP was performed with a second antibody. When ChIP was first performed with anti-HBZ, sequential ChIP showed occupancy of Sp1 in the same protein-DNA complex (Fig 5C, lane 6). Alternatively, when ChIP was first performed with anti-JunD, sequential ChIP showed occupancy of Sp1 in the same protein-DNA complex (lane 5). Specificity was again demonstrated, as these complexes were not detected at the distal region of the hTERT promoter

**Figure 6:**
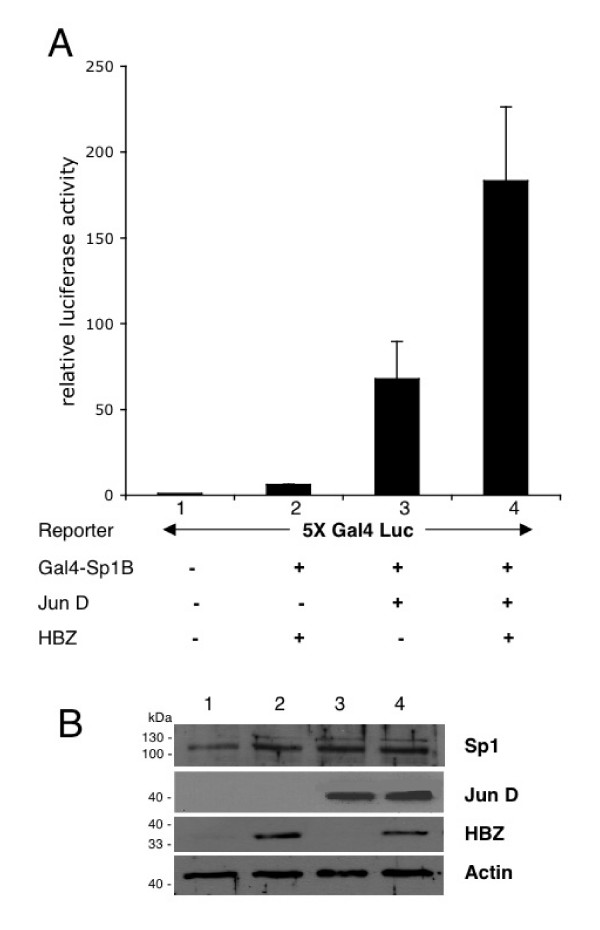
Functional interactions between HBZ, JunD and Sp1. (A) HeLa cells were cotransfected with the reporter construct 5XGal4-Luc (200 ng) consisting of five tandem Gal4-binding sites in the absence (-) or presence (100 ng) of expression vectors for Gal4-Sp1B (containing the B domain of Sp1), and/or HBZ and JunD. Luciferase activity was normalized to tk-luc activity. Results represent duplicate samples from three different experiments. (B) Western blot analysis of Sp1, HBZ and JunD protein levels in whole cell lysates of HeLa samples. The membrane was successively probed with rabbit polyclonal anti-Sp1, anti-HBZ sera, and mouse monoclonal anti-Flag and anti-actin antibodies.

Additional experiments were next performed to establish functional interactions between JunD, HBZ and Sp1. Transient co-transfection experiments in HeLa cells showed that JunD transactivated a synthetic promoter consisting of five tandem high affinity binding sites for the yeast protein Gal4 upstream of a minimal TATA box (5XGal4-Luc) by 11-fold (Fig 6, compare lanes 3 and 2), when it was coexpressed with a Gal4-Sp1B chimeric protein consisting of the DNA binding domain of Gal4 fused to the B domain of Sp1, indicating that JunD and Sp1 are able to interact physically. Overexpression of HBZ in HeLa cells enhanced the JunD-mediated transactivation potential by 2.7-fold (compare lanes 3 and 4). Control experiments showed that the HBZ protein alone in the presence of Gal4-Sp1B had no significant effect on the activity of the 5XGal4 promoter (lane 2). These observations show that cooperative interactions between HBZ, JunD and Sp1 can transactivate promoters containing multiple Sp1-binding sites. Collectively, these results clearly underline that the synergistic transactivation of hTERT promoter by HBZ and JunD is Sp1-dependent.

### Effect of Tax on HBZ-mediated activity of the hTERT promoter

**Figure 7:**
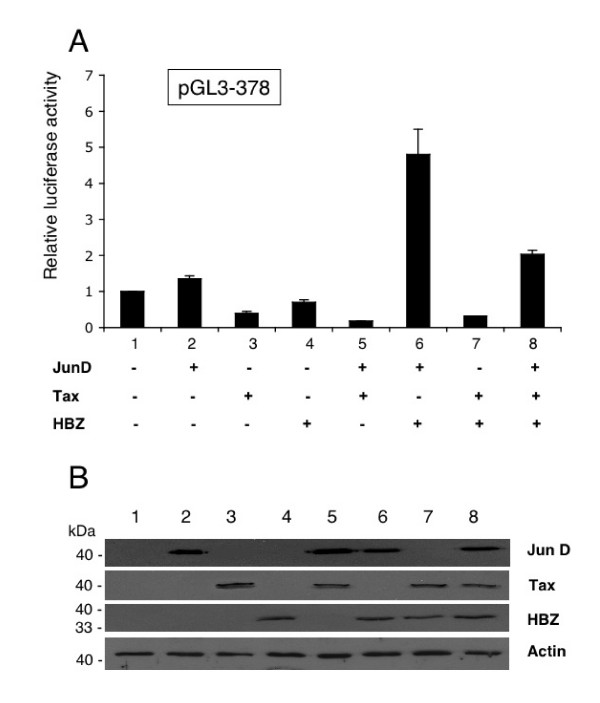
Effect of Tax on HBZ-mediated activation of the hTERT promoter. (A) The luciferase reporter plasmid, pGL3-378 (100 ng) was cotransfected with expression plasmids for HBZ (200 ng), JunD (200 ng) and Tax (10 ng), as indicated. Luciferase activity, normalized to tk-luc activity, is presented relative to that of cells transfected with the reporter plasmid alone. Results represent triplicate samples, from three different experiments. (B) Western blot analysis of HBZ, Tax and JunD protein levels in whole cell lysates of HeLa samples. The membrane was successively probed with a rabbit polyclonal anti-HBZ, anti-Tax sera, mouse monoclonal anti-Flag and rabbit anti-actin antibodies.

It has been previously shown that Tax acts as a transcriptional repressor of the hTERT core promoter through the proximal E-box, by competing with cMyc/Max bHLH proteins for recruitment of the CBP/p300 co-activators [26]. In addition, we have observed a down-regulation of hTERT transcription by Tax in HTLV-1 transformed or in Tax-expressing T lymphocytes [27]. Our present results therefore propose that Tax and HBZ may exert opposite effects on the activity of the core promoter. To verify this possibility, HeLa cells were co-transfected with the reporter pGL3-378 construct together with the Tax-, HBZ- and JunD-expression vectors (Fig 7). As expected, the basal transcriptional activity of the core promoter was repressed by Tax (lane 3). The co-expression of JunD had no effect on the repression exerted by Tax (lane 5). As expected, the basal activity was enhanced by 5-fold, when HBZ and JunD were expressed (lane 6); however, only a 2-fold activation was observed when HBZ, JunD and Tax were co-transfected (lane 8). These data provide a clear indication that the HTLV-1 encoded proteins, HBZ and Tax, exert antagonistic effects on the transcription of the *hTERT *gene. They further propose that HBZ is fully active only when Tax is silenced.

## Discussion

The novel viral HBZ protein coded in the minus strand of the HTLV-1 provirus has been shown to display a bimodal RNA- and protein-based function (see [16,28] for reviews). Indeed, *HBZ *RNA was found to be implicated in the proliferation of infected cells [8]. The protein, through its interactions with AP-1 proteins acquires the ability to intervene, in the regulation of viral and cellular gene transcription [10,14,15]. Thus, studies performed in HeLa cells with synthetic or natural promoters containing AP-1 consensus sites have indicated that HBZ inhibits the transcriptional activation mediated by c-Jun, while it enhances the activity of JunD [11-13]. Here, we demonstrate that the HBZ protein behaves as a positive or negative regulator of the hTERT promoter depending on the Jun partner. Indeed, HBZ together with JunD activates hTERT transcription, whereas HBZ with c-Jun represses it. We also observe a significant increase of hTERT transcripts in cells expressing HBZ and JunD, in spite of the inhibitory effects exerted by AP-1 proteins on the distal regions of the hTERT promoter [23]. To our knowledge, the present study is the first that describes the effect of HBZ on a cellular gene expressed in tumour cells.

We also report that HBZ and JunD target the proximal region of the promoter in which no AP-1 site is present. Consequently, the activity of HBZ/JunD is independent of the DNA-binding properties of JunD, but instead requires the interaction of these bZIP factors with other nuclear factors. The proximal 180 bp of the hTERT core promoter is important for maintaining basal transcriptional activity of which c-Myc/Max and Sp-1 are the main activators [21-23]. Previous studies clearly demonstrated that c-Jun and related proteins (JunB, JunD and ATF-2) cooperate with Sp1 to transactivate the promoter of the human p21 gene by acting as a superactivator of the Sp1 transcription factors [24,25]. We have therefore hypothesized that these factors together with HBZ and JunD are involved in the activation of the hTERT promoter. We have indeed found that co-expression of JunD and HBZ resulted in a strong synergistic transactivation of a luciferase reporter construct consisting of one Sp1 consensus site upstream of a TATA-box. We have further shown by immunoprecipitation and ChIP assays that the HBZ-JunD heterodimers are tethered to the proximal hTERT promoter via interaction with Sp1. Consequently, we propose that HBZ plays a positive role on hTERT transcription by cooperating with JunD, in an indirect manner through Sp1 transcription factors. Indeed, we have recently demonstrated that HBZ possesses a modulatory domain immediately adjacent to its bZIP domain involved in the stimulation of JunD transcriptional activity [29]. This domain would influence the conformational structure of the AP-1 heterodimers to form a complex with more accessibility to the transcriptional regulators [30,31].

Various viral proteins have also been implicated in Sp1-dependent cellular gene transcription. For instance, the oncoprotein v-Jun downregulates *SPARC *and *collagenase alpha2(I) *transcription through the formation of a DNA-Sp1-v-Jun chromatin-associated complex [32,33]. The E1A tumor suppressor protein of adenovirus upregulates hTERT transcription through Sp1 binding sites, involving recruitment of p300/CBP proteins [34]. Finally, the activity of the proximal promoter of hTERT is upregulated by the interaction of Sp1 with the latency-associated nuclear antigen (LANA), which potentially contributes to the immortalization of Kaposi's Sarcoma-associated herpes virus-infected cells [35]. These data support the hypothesis for Sp1-binding sites in hTERT promoter as the responsive sequences to the Sp1-JunD-HBZ complexes.

Our experimental strategy to apprehend the role of HBZ on hTERT transcriptional regulation was based upon transient transfection assays performed in HeLa cells. These cells, which are widely used in studies on the transcriptional regulation of gene expression, have been shown to display a moderate transcriptional activity of hTERT, when compared to other cancer cell lines and to normal cells. Furthermore, a close correlation has been observed between Myc and Sp1 expression and levels of hTERT transcriptional activity [21]. Last but not least, these cells were found to support the constitutive expression of HBZ, after transduction with a bicistronic retrovirus coding for HBZ and the green fluorescent marker GFP, contrary to T cells lines, such as CEM and Jurkat (data not shown).

**Figure 8:**
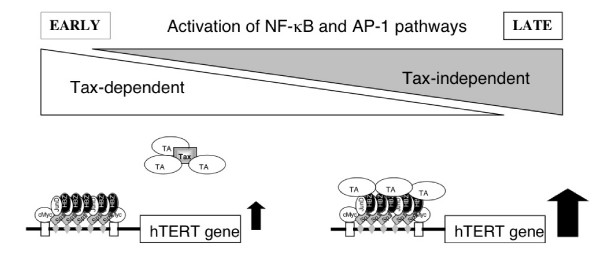
Proposed model for the transcriptional regulation of the hTERT core promoter during the HTLV-1-induced leukemic process (see Discussion). The early steps of the leukemic process during which HTLV-1 infected T-lymphocytes were expressing Tax and HBZ are characterized by the recruitment of transcriptional activators (TA) by Tax. Later, Tax expression is decreased and/or silenced and the TA become available to interact with HBZ/JunD complexes. In both cases, HBZ/JunD complexes are interacting with the Sp1 transcription factor bound to the GC boxes present in the hTERT core promoter.

Taken into account our present observations together with the relevant published data, a model for the regulation of the hTERT proximal region of the promoter during the multistep process of leukemogenesis is proposed (Fig 8). During the early steps of the transformation of T lymphocytes infected by HTLV-1, Tax is playing a pivotal role, by increasing the transcription of numerous cellular genes, and specially that of NF-κB and AP-1 [28]. In these activated T cells, Tax has been shown to repress the hTERT core promoter activity [26,27,36]. Late in ATL, Tax expression is decreased and/or silenced. Indeed, leukemic cells are selected among cells that carry deletions in the proviral DNA and in which the *tax *gene is not expressed [37]. As HBZ is the only viral gene product detected in ATL cells [8], our present observations clearly infer that concomitantly with the loss of Tax, HBZ becomes fully responsible in the increase of hTERT transcription observed during the late stages of leukemogenesis. Now, the telomerase activity in chronic ATL patients was found to be higher than that in HTLV-1 carriers and healthy donors. Furthermore, the reactivation of telomerase in peripheral blood mononuclear cells of ATL patients has been shown to provide a good marker to predict worsening of ATL, especially during the evolution from the chronic to the acute type [4,38]. Finally, the presence of significant shorter telomeres in chronic ATL patients, compared to those of the two other subject groups, is pleading for a telomeric dysfunction. Such an event might favor a genetic instability that would be perpetuated through an increase of hTERT transcription, to which HBZ is participating through its interactions with JunD and Sp1.

## Methods

### Plasmids

hTERT promoter-luciferase reporter constructs (pGL3-3300, pGL3-2000 and pGL3-378) used in this study have been previously described [23]. The reporter plasmid, pGL2-Sp1-TATA-Luc, contains a single copy of Sp1 binding site fused with a TATA box and the luciferase gene [33]. The pCMV-Tax expression vector was obtained from Dr. W.C. Greene (USA). pcDNA-HBZ-Myc encoding the SI isoform of HBZ and the mutated versions (HBZΔAD, HBZΔbZip and HBZΔADΔZip) were previously described [9,10]. The AP-1 expression vectors pcDNA-c-Jun et pCMV-JunD-Flag were obtained from Dr. M. Piechaczyk (Montpellier, France). The Sp1 expression plasmid, pMIC-Sp1, was a generous gift of Dr. J. Marvel (Lyon, France). The Gal4-Sp1B construct containing the Gal4 DNA binding domain portion (aa 1-170) fused to the domain B of Sp1 (aa 263-542) was a generous gift of Dr. D. Kardassis (Heraklion, Greece). The 5XGal4-Luc reporter (pG5Luc) containing five tandem binding sites for the yeast protein Gal4 upstream of a minimal TATA box was purchased from Promega, France.

### Cells, transfections, luciferase and Western blot assays

HeLa cells were grown in Dulbecco's modified Eagle's medium (Invitrogen Life technologies, Frederick, MD) supplemented with 10% heat-inactivated fetal calf serum and 100 IU/ml penicillin, 50 μg/ml streptomycin at 37°C in a 5%CO2 atmosphere. They were transfected using the calcium phosphate precipitation method [39]. Jurkat lymphoblastoid T cells were grown in complete RPMI 1640 medium (Invitrogen) and were transfected by electroporation at 250 V and 950 μFd with a Cellject electoporator (Eurogentec). Transfection efficiencies were normalized by cotransfection of a Renilla expression vector (RL-TK, Promega). Assays were performed 30 h after transfection using the dual Luciferase Reporter assay (Promega) and a Berthold luminometer. Western blots were performed using 3 μg of protein lysates, and were revealed by using polyclonal HBZ antibody [5], polyclonal Sp1 antibody (generous gift of J. Marvel, Lyon, France). Mouse monoclonal anti c-myc antibodies were purchased from Roche, anti-Flag and anti-actin (AC-40) antibodies were purchased from Sigma. Secondary HRP-linked antibodies were purchased from Immunotech (France). Blots were developed using an enhanced chemiluminescence detection system (Renaissance, NEN Life Science Products). Bands were visualized by using Hyperfilm (Amersham Pharmacia Biotech).

### Immunoprecipitation and *in vivo *interaction of HBZ, JunD and Sp1

HeLa cells (0.7 × 10^6 ^cells/10-cm dishes) were transfected with 5.5 μg of each plasmids (pcDNA-HBZ-Myc, pCMV-JunD-Flag and pMIC-Sp1) using the calcium phosphate precipitation method. 48 h post-transfection, cells were lysed in IP buffer (50 mM Tris pH8, 150 mM NaCl, 0.5% Nonidet-P40) supplemented with complete protease inhibitors (Roche Diagnostics). Cell lysates were centrifuged at 12,000 g for 10 min at +4°C. Equal amounts of cell lysates (400 μg) were first incubated with a control serum to preclear the lysate. Precleared lysates were then incubated with anti-HBZ polyclonal antibody overnight at +4°C with rotation and further incubated with protein G Plus/ProteinA Agarose beads (Calbiochem) at +4°C for 30 min with rotation. The immunoprecipitated complexes were washed five times with 0.5 ml of ice-cold IP buffer. The immunoprecipitated pellets were resuspended in 20 μl of 2X-SDS protein sample buffer and then resolved on 10% SDS-PAGE and detected by Western blot assay using anti-Flag and anti-Sp1 antibodies.

### RT-PCR and quantitative real-time PCR (qPCR)

Total RNAs were isolated from cells using Trizol reagent (Invitrogen) according to the manufacturer's instructions. Samples were treated with RNase-free DNase (10 U/μl, Qiagen) for 30 min at 20°C and then for 15 min at 65°C. Five-hundred ng of RNA were reverse transcribed by using oligo(dT)12-18 and Superscript II (InVitrogen). Reverse transcription was performed for 50 min at 42°C. The total cDNA (20 μl) was frozen until PCR was performed. After thawing, 2 μl of cDNA diluted in distilled water were used for each PCR reaction. The real-time quantitative PCR (qPCR) was performed in special lightcycler capillaries (Roche) with a lightcycler Instrument (Roche), by using the Platinium SYBR-Green qPCR SuperMix UDG kit (Invitrogen). The following specific primers were used to detect: hTERT sense, 5'-TGTTTCTGGATTTGCAGGTG-3' and antisense, 5'-GTTCTTGGCTTTCAGGATGG-3', actin sense, 5'-TGAGCTGCGTGTGGCTCC-3' and antisense: 5'-GGCATGGGGGAGGGCATACC-3'. The following program was used: samples were incubated at 50°C for 2 min, followed by 10 min at 95°C, followed by 40 cycles (95°C 10 sec, 61°C 5 sec, 72°C 10 sec). The dissociation curve was measured for each sample. Relative level of hTERT sequence against the reference actin sequence was calculated using the ΔC_t _method. A standard calibration curve was performed, using cDNA from HeLa cells. The levels of actin transcripts were used to normalize the amount of cDNA in each sample.

### Chromatin immunoprecipitation (ChIP) assay *in vivo*

ChIP assays were performed essentially by using the Upstate Biotechnology Inc. recommendations with minor modifications. Formaldehyde cross-linked chromatin from 5 × 10^6 ^cells/antibody was used for each immunoprecipitation. Cross-linking reactions were quenched with 125 mM glycine, cells were lysed, and chromatin was sonicated to obtain an average DNA length of 500 bp. Following centrifugation, the chromatin was diluted 10-fold and pre-cleared with protein A-agarose containing salmon sperm DNA and bovine serum albumin (Upstate Biotechnology). Pre-cleared chromatin (2 ml) was incubated overnight at +4°C with 5 μg of antibody recognizing JunD (sc-74, Santa Cruz Biotechnology) or Sp1 (sc-70, Santa Cruz Biotechnology) or with 10 μl of anti-HBZ or normal rabbit serum, followed by protein A-agarose immunoprecipitation. Eluted protein-DNA cross-links were reversed by heating at 65°C overnight, and 25% of the recovered DNA was used in PCR reaction to amplify the 278-bp region of the hTERT proximal promoter with the Phusion high-fidelity DNA polymerase (Ozyme) enzyme and the forward primer (-190/-171) 5'-CACAGACGCCCAGGACCGCG-3' and the reverse primer (+69/+88) 5'-GCGCGCGGCATCGCGGGGGT-3'. A control PCR (negative control leading to a 150-bp fragment) was also performed from a region devoided of Sp1 or AP1 sites within the distal region of hTERT using forward primer (-2916/-2897) 5'-GGCAGGCACGAGTGATTTTA-3' and reverse primer (-2782/-2763) 5'-CTGAGGCACGAGAATTGCTT-3' to show the specificity of Sp1 sites located on the proximal hTERT in the ChIP assay. DNA samples recovered from chromatin samples before immunoprecipitation, which corresponds to 1% of chromatin samples included in each immunoprecipitation reaction, were also PCR amplified as loading controls. The PCR reactions for hTERT were processed through 32 cycles of 98°C for 10 sec and 71°C for 30 sec, followed by one cycle for 7 min at 72°C. PCR products were separated on 2% agarose gel and visualized with ethidium bromide staining.

For sequential ChIP assays, the initial ChIP was performed with the indicated antibodies, the primary immunocomplex was then eluted by 10 mM dithiothreitol at 37°C for 30 min. The eluate was diluted 50 times with buffer (20 mM Tris-Hcl, pH8.1, 150 mM NaCl, 2 mM EDTA, and 1% Triton X-100) and a second ChIP was then carried out.

### Statistical analysis

Data were expressed as mean ± SD, and when required were compared by one-way ANOVA with Dunnett's test, P < 0.05, was taken as statistically significant.

## Competing interests

The author(s) declare that they have no competing interests.

## Authors' contributions

ASK carried out most of the transfection experiments, luciferase assays and western blot analyses and drafted the manuscript. JV has initiated the ChIP assays. MDD has conducted the RT-PCR and IP analyses. MC has participated in the design of the experiments concerning the identification of the Sp1 binding sites. JMM has helped in finalizing the manuscript and has provided important input on the design of the study. LG and MDD have conceived the study, participated in its coordination, helped in drafting and finalizing the manuscript. All authors have read and approved the final manuscript.
